# Negative PSMA PET can be used to avoid unnecessary pelvic lymph node dissection in intermediate risk prostate cancer

**DOI:** 10.1038/s41391-024-00930-z

**Published:** 2025-01-06

**Authors:** Reha-Baris Incesu, Felix Preisser, Florian Nohe, Tobias Maurer, Markus Graefen, Derya Tilki

**Affiliations:** 1 Martini-Klinik Prostate Cancer Center, University Hospital Hamburg Eppendorf, Hamburg, Germany; 2 Department of Urology, University Hospital Hamburg-Eppendorf, Hamburg, Germany; 3 Department of Urology, Koc University Hospital, Istanbul, Turkey

**Keywords:** Cancer therapy, Prostate cancer

## Abstract

**Background:**

Prostate-specific membrane antigen (PSMA) positron emission tomography (PET) has a high negative predictive value (NPV) in determining lymph node invasion (LNI) in men with intermediate-risk disease undergoing radical prostatectomy (RP) and pelvic lymph node dissection (PLND). We hypothesized that PSMA PET may be used to reduce the number of unnecessary PLND procedures performed.

**Objective:**

To assess BCR-free survival of intermediate risk prostate cancer patients with a negative PSMA PET who underwent PLND vs. no PLND.

**Design, setting, and participants:**

Within a high-volume center database, we identified patients with Grade Group 2-3 and PSA ≤ 20 ng/ml who had a negative PSMA PET prior to RP between 2016 and 2021.

**Outcome measurements and statistical analysis:**

Kaplan–Meier analyses were used to compare BCR-free survival between patients with and without PLND.

**Results and limitations:**

Overall, 371 patients were identified. Of those, 333 (90%) underwent RP with PLND, while 38 (10%) had no PLND during RP. Median number of removed lymph nodes in patients with PLND was 16. The NPV of PSMA PET for LNI detection was 90.1%. Median follow-up was 36 months. The median preoperative PSA was 7.8 ng/ml. 59% had biopsy Grade Group 2 and 41% had biopsy Grade Group 3, respectively. BCR-free survival at 36 months after prostatectomy was 78.7% vs. 76.7% (*p* = 0.8) for patients with vs. without PLND. Main limitation is the absence of long-term oncologic outcomes.

**Conclusions:**

In intermediate risk prostate cancer patients, a PLND may be avoided in the presence of a negative PSMA PET.

## Introduction

Pelvic lymph node dissection (PLND) during radical prostatectomy (RP) is often performed as nodal staging procedure in intermediate prostate cancer (PCa) patients. However, a significant proportion of patients undergoing PLND don’t harbor lymph node invasion (LNI) at final pathology. Moreover, PLND is associated with higher risk of complications, such as symptomatic lymphocele, lymphedema, nerve injury, or thromboembolic events [1]. In consequence, it is of utmost importance to optimize the indication for PLND during RP. Diagnostic tools such as nomograms can help to select the right patients for PLND, but only to a limited extend. Moreover, limited sensitivity and specificity of conventional imaging impede reliable imaging-based decisions. Conversely, new imaging tools such as prostate-specific membrane antigen (PSMA) positron emission tomography (PET) have reportedly a higher diagnostic accuracy. Specifically, a high negative predictive value (NPV) has been reported for PSMA PET in detecting LNI in men with intermediate-risk disease undergoing PLND during RP [2]. However, it is unknown if patients that are spared of PLND based on a negative PSMA PET suffer worse oncologic outcomes compared to patients with PLND despite a negative PSMA PET. We addressed this void and hypothesized that negative PSMA PET/CT may be used to reduce the number of unnecessary PLND procedures performed. We tested this hypothesis assessing biochemical recurrence (BCR)-free survival of intermediate risk prostate cancer patients with a negative PSMA PET who underwent PLND vs. no PLND.

## Patients and methods

### Patients

After institutional review board approval, 371 patients with available PSMA PET data for primary staging who underwent RP with or without PLND between 2016 and 2021 at the Martini-Klinik with a biopsy Grade Group 2 or 3 and PSA ≤ 20 ng/ml were identified. Only patients with a PSMA PET indicating negative results for LNI were included. Exclusion criteria consisted of missing follow-up prostate-specific antigen (PSA) values. RP was performed using an open retropubic (*n* = 134, 36%) or robot-assisted approach (*n* = 237, 64%) [3–5]. PSMA PET scans for primary staging were performed at the discretion of the treating physician. These scans corresponded to single primary staging methodology in all patients included and were performed before surgery.

### PSMA PET scans

PSMA PET scans were performed in nuclear medicine institutions throughout Germany. 68Ga or 18 F was used as tracer for PSMA PET scans. The median injected activity of the tracers was 185 MBq (IQR: 140-252.8 MBq). PET scans were performed in combination with either computer tomography (native, low-dose or contrast; 95%) and/or MRI (5%).

### BCR-free survival

BCR was defined as two consecutive prostatic specific antigen (PSA) values ≥0.2 ng/ml after surgery. PSA follow-up was performed postoperatively by treating urologists. PSA values were obtained individually from patients by the Martini-Klinik. BCR-free survival for each patient was recorded in months, starting from the date of surgery until the date of BCR.

### Statistical analyses

All analyses stratified the study population between patients that underwent PLND vs. no PLND. Within descriptive analyses, the Kruskal-Wallis rank sum test was used for differences in median values and Pearson’s Chi-square test for differences in proportions. Kaplan–Meier plots depicted BCR-free survival. In all statistical analyses, R software environment for statistical computing and graphics (The R Foundation for Statistical Computing, Vienna Austria, R version 4.2.1) was used [6]. All tests were two-sided, with a significance level set at *p* < 0.05.

## Results

### Descriptive characteristics

We identified 371 prostate cancer patients presenting a negative PSMA PET for primary staging before RP (Table 1). Median age was 65 years (interquartile range [IQR] 59–69 years). Median follow-up was 36 months. Median preoperative PSA was 7.8 ng/ml (IQR 5.1–11.7 ng/ml). Of all patients, 220 (59%) had biopsy Grade Group 2 and 153 (41%) had biopsy Grade Group 3, respectively. Moreover, of 371 patients, 239 (64%) were treated with the robotic approach vs. 134 (36%) who were treated with an open retropubic approach. At final pathology, 12 (3%) patients presented with Grade Group 4-5 vs. 357 (96%) with Grade Group 2-3.

**Table 1:** Baseline characteristics of 371 prostate cancer patients after radical prostatectomy, within the institutional database (2016–2021), stratified according to patients with PLND vs. without PLND.

Characteristic	All patients (*n* = 371)	PLND (*n* = 333)	No PLND (*n* = 38)	*p*-value
Median age at surgery, yrs (IQR)	65 (59–69)	65 (59–70)	64 (59–69)	0.7
Median PSA at diagnosis, ng/ml (IQR)	7.8 (5.1–11.7)	7.8 (5–11.8)	7.6 (5.7–10.2)	0.9
Median number of positive biopsy cores (IQR)	5 (3–8)	5 (3–8)	4 (2.2–7)	0.4
Median number of removed lymph nodes (IQR)	–	16 (10–22)	–	–
Pathologic lymph node status, *n* (%)				
N0	300 (80.4)	300 (90.1)	–	–
N1	33 (8.8)	33 (9.9)	–	–
NX	38 (10.2)	–	38 (100)	–
Biopsy Grade Group, *n* (%)				
2	220 (59)	191 (57.4)	28 (73.7)	0.1
3	153 (41)	142 (42.6)	10 (26.3)	
Surgical approach, *n* (%)				
Robotic-assisted	239 (64.1)	213 (64)	24 (63.2)	1.0
Open retropubic	134 (35.9)	120 (36)	14 (36.8)	
Specimen Grade Group, *n* (%)				
1	2 (0.5)	2 (0.6)	0 (0)	0.4
2	249 (66.8)	219 (65.8)	30 (78.9)	
3	108 (29)	101 (30.3)	7 (18.4)	
4-5	12 (3.2)	11 (3.3)	1 (2.6)	
Pathologic T stage, *n* (%)				
pT2	194 (52)	168 (50.5)	26 (68.4)	0.1
pT3a	120 (32.2)	112 (33.6)	8 (21.1)	
≥pT3b	57 (15.3)	53 (15.9)	4 (10.5)	
Nerve sparing, *n* (%)				
Bilateral	285 (76.4)	254 (76.3)	29 (76.3)	1.0
None	21 (5.6)	19 (5.7)	2 (5.3)	
Unilateral	67 (18)	60 (18)	7 (18.4)	
Surgical margins, *n* (%)				
Negative	299 (80.2)	267 (80.2)	32 (84.2)	0.6
Positive	72 (19.3)	66 (19.8)	6 (15.8)	
Adjuvant radiotherapy performed, *n* (%)	18 (4.8)	17 (5.1)	1 (2.6)	0.2

*IQR* interquartile range, *PLND* pelvic lymph node dissection, *PSA* prostate-specific antigen, *RP* radical prostatectomy.

Of 371 patients, 333 (90%) underwent PLND vs. 38 (10%) who had no PLND during RP. Median number of removed LN in patients with PLND was 16 (IQR 10–22).

### Diagnostic performance of PSMA PET/CT

Of 333 patients with PLND and a negative PSMA PET, 33 (10%) had LNI (pN1) vs. 300 (90%) had no LNI (pN0). With regard to diagnostic performance PSMA PET in LNI detection, 33 (10%) were identified as false-negative vs. 300 (90%) were identified as true-negative. In consequence, the NPV of a PSMA PET for LNI detection was 90%. Median diameter of tumor involvement in positive lymph nodes within the 33 patients with LNI was 2.2 mm.

### BCR-free survival in patients with PLND vs. no PLND

**Fig. 1:**
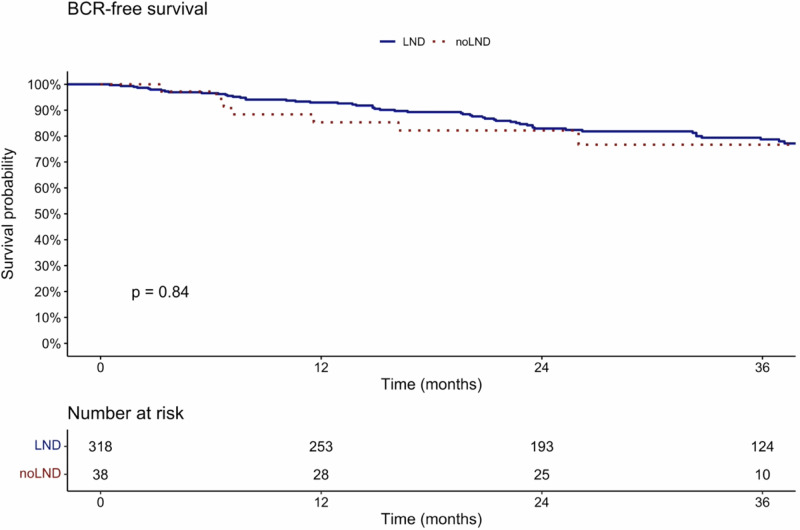
Kaplan–Meier plots illustrating BCR-free survival in prostate cancer patients after radical prostatectomy, with negative preoperative PSMA-PET CT, stratified according to patients with PLND vs. without PLND.

In Kaplan–Meier analyses, BCR-free survival at 36 months after prostatectomy was 78.7% vs. 76.7% (*p* = 0.8) for patients with vs. without PLND (Fig. 1). Within the follow-up 22.1% of patients developed a BCR.

## Discussion

PLND is the gold standard of nodal staging in men with newly diagnosed intermediate risk prostate cancer. However, it represents a complicative procedure without a clear oncological benefit for this patient subgroup [7]. Therefore, new promising imaging modalities such as PSMA PET might help to select the right patients for PLND during RP. However, it is unknown if it is safe to spare PLND based on a negative PSMA PET. We addressed this void and hypothesized that BCR-free survival might not be different in intermediate risk prostate cancer patients with a negative PSMA PET who underwent PLND vs. no PLND. We relied on a high-volume single institution database and made several noteworthy findings.

First, within the current study, we identified 371 prostate cancer patients with a Grade Group of 2 or 3 that had a negative PSMA PET before undergoing RP with or without PLND. Of those, 333 (90%) underwent PLND. The use of PSMA PET for primary staging in prostate cancer remains limited [8]. Previous reports on PSMA PET as primary staging modality in prostate cancer did not focus exclusively on intermediate risk patients and patients with a clinical negative nodal status at PSMA PET [9–11]. Therefore, our observations cannot be directly compared to other reports. Despite our homogeneous patient selection criteria, sample size of the current study was good when compared to previous analyses that included patients of various risk categories or patients with both negative and positive clinical nodal status, at PSMA PET [2].

Second, no statistically significant differences in baseline characteristics were identified between patients with PLND vs. without PLND. Patients with PLND exhibited a higher rate of biopsy Grade Group 3. However, the lack of statistical significance corroborates the comparability of both patient groups (PLND vs. no PLND).

Third, of 333 patients undergoing PLND, 33 (10%) harbored LNI. The rate of LNI is lower than described in previous studies (14.5–36.6% [12–15]), that included varying proportions of intermediate and high-risk patients. Conversely, our study relied exclusively on patients with a Grade Group 2-3 and PSA < 20 ng/ml. It is known that intermediate-risk patients have a lower risk of LNI than high-risk patients [16]. This could explain the lower rate of LNI in the current study. Finally, our median number of removed LN was 16 (IQR 10–22). This number is within the range of previous analyses that exhibited numbers of removed LN 11–24 [2]. In consequence, the current study provides a representative group of intermediate risk patients that underwent PLND during RP.

Fourth, there is a lack of studies comparing oncologic outcomes between miN0 patients that underwent RP with PLND vs. without PLND. Specifically, BCR-free survival at 36 months after prostatectomy was 78.7% in patients with PLND vs. 76.7% in patients without PLND (*p* = 0.8). In consequence, our study indicates that patients without PLND have similar BCR-free survival at 36 months after RP when compared to patients with PLND. This finding is important given the ongoing discussions about PLND at the time of RP. Previous studies assessed BCR-free survival in PLND patients: For example, Amiel et al. demonstrated that pN1 patients with positive LNI status in PSMA PET/CT (miN1) exhibited lowest BCR-free survival, followed by pN1 patients with negative LNI status in PSMA PET/CT (miN0), followed by pN0 miN0 patients (BCR-free survival: pN1 miN1 < pN1 miN0 < pN0 miN0) [17]. Moreover, in another report the authors compared exclusively pN1 patients and confirmed a lower median BCR-free survival in pN1 miN1 patients vs. pN1 mi0 patients (7.9 months vs. 13.7 months, *p* = 0.006) [18]. These studies confirm that preoperative detection of positive LN (miN1) in PSMA PET, compared to no detection of positive LN (miN0) is associated with worse oncological outcomes. These studies help to determine which patient groups within those that received PLND, are at higher risk of worse survival and therefore should be monitored closely after surgery. Conversely, the findings of the current study have an impact on clinical decision-making before surgery. Specifically, these findings suggest that PSMA PET/CT negative patients might not benefit from a PLND. In consequence, the complication-prone procedure of PLND could be spared in selected intermediate risk patients with a PSMA PET negative for LNI.

Finally, within the PLND patients of the current study, 10% of the patients harbored LNI despite a negative PSMA PET. However, the median diameter of patients with LNI was only 2.2 mm. In consequence, it cannot be ruled out that a similar proportion of LNI exists within the patients that did not receive PLND. However, given the high sensitivity of PSMA PET in detecting LN metastases [19], a reason for a false-negative PSMA PET results might be small size of LN metastases. The fact that patients with PLND vs. no PLND exhibited no difference in BCR-free survival, points to the possibility that missed LN metastases do not affect short-term clinical outcome of patients without PLND. Indeed, a recent study indicated that patients with a smaller diameter of the largest nodal metastasis had a significantly lower risk of BCR after RP [18].

Taken together, we present new insights in patients with negative nodal status at PSMA PET, where the performance of PLND does not affect short-term oncologic outcome. Specifically, BCR-free survival was not different after 36 months between patients that underwent RP with PLND vs. without PLND. It is noteworthy that a non-negligible number of patients with clinically negative (miN0) lymph node status had pathological positive (pN1) nodal status and that PLND did not change short-term oncologic outcome in these patients.

Despite its novelty, the current study has several limitations. First, the study is limited by its retrospective design. Second, PSMA PET scans were performed in multiple centers in Germany without superior coordination and without a central review. Third, our cohort included both 68Ga and 18 F as tracers which could limit the comparability of imaging results. Fourth, distribution trends of adverse clinical and tumor characteristics existed in the study cohort. Specifically, patients with PLND exhibited a higher rate of ≥pT3a, positive margins as well as a higher rate of Grade Group ≥3 in final pathology. In consequence, a selection bias, where PLND may have been more likely performed in patients with adverse characteristics, cannot be ruled out. However, the lack of statistical significance in these observed trends corroborates the comparability of both patient groups (PLND vs. no PLND). Patients with intermediate risk disease were usually treated with RP and PLND at our institution during the study period. Fifth, we could only assess BCR-free survival and no long-term oncologic outcomes like metastases-free or cancer-specific survival. This could provide a more appropriate prognosis of patients’ outcome. Finally, our median follow-up of 36 months represents a limitation by itself. Nonetheless, our cohort size, the performance of surgery within a high-volume center by only experienced surgeons as well as the coordinated processing of the histological specimen by one experienced pathological department display the strengths of this study.

## Conclusions

In intermediate-risk prostate cancer patients with negative nodal status at PSMA PET, the performance of PLND does not affect short-term oncologic outcome. Therefore, in these patients, a PLND may be avoided in the presence of a negative PSMA PET.

## Data Availability

The datasets and statistical codes generated during and/or analyzed during the current study are available from the corresponding author on reasonable request.
